# Triglyceride‐Related Indices and Risk of Acute Pancreatitis: A Prospective Study in UK Biobank

**DOI:** 10.1002/kjm2.70189

**Published:** 2026-03-10

**Authors:** Jing‐Bo Li, Yu‐Jiao Zhu, Yang‐Jie Liao, Jia‐Yi Wang

**Affiliations:** ^1^ Department of Gastroenterology, the Third Xiangya Hospital Central South University Changsha China; ^2^ Department of Gastroenterology, Changde Hospital, Xiangya School of Medicine, Central South University (The First People's Hospital of Changde City), Changde China

**Keywords:** acute pancreatitis, cohort study, triglyceride‐related indices, UK biobank

## Abstract

Triglyceride‐related indices, including triglyceride‐glucose (TyG), TyG related to body mass index (TyG‐BMI), TyG related to waist circumference (TyG‐WC), TyG related to waist‐to‐height ratio (TyG‐WHtR), atherogenic index of plasma (AIP), cardiometabolic index (CMI), visceral adiposity index (VAI), and lipid accumulation product (LAP), are simple surrogate indicators of insulin resistance. Nevertheless, few studies have explored the association between triglyceride‐related indices and acute pancreatitis (AP). A total of 387,275 participants from the UK Biobank were included. Cox regression models provided hazard ratios (HRs) with 95% confidence intervals (CIs) for the association between triglyceride‐related indices and the risk of AP. Restricted cubic splines (RCS) and receiver operating characteristic (ROC) were employed to evaluate and visualize the relationship. These triglyceride‐related indices were significantly associated with an increased risk of AP. Compared with the lowest quartile, the HRs (95% CIs) of AP in the highest quartile were 1.52 (1.33, 1.74), 1.54 (1.34, 1.77), 2.32 (2.02, 2.65), 2.83 (2.43, 3.30), 2.86 (2.46, 3.32), 2.16 (1.88, 2.49), 1.95 (1.71, 2.22), and 2.49 (2.15, 2.87), respectively, for the TyG, AIP, TyG‐BMI, TyG‐WC, TyG‐WHtR, CMI, VAI, and LAP index. RCS analyses showed that the above relationships except for AIP were nonlinear (P for nonlinear < 0.05). ROC curves showed that TyG‐WHtR was the best predictor for AP. Elevated triglyceride‐related indices were positively related to AP onset. Among them, the TyG‐WHtR index displayed the strongest predictive power for the onset of AP, which was expected to become a more effective metric for identifying populations at early risk of AP.

**Abbreviations**
AIP: Atherogenic index of plasma
AP: Acute pancreatitis
AUC: Area under curve
BMI: Body mass index
CIs: Confidence intervals
CMI: Cardiometabolic index
CRP: C‐reactive protein
FBG: Fasting blood glucose
HDL‐C: High‐density lipoprotein cholesterol
HOMA‐IR: Homeostatic model assessment for insulin resistance
HRs: Hazard ratios
ICCs: Intraclass correlation coefficients
IQR: Interquartile range
IR: Insulin resistance
LAP: Lipid accumulation product
RCS: Restricted cubic splines
ROC: Receiver operating characteristic
SAP: Severe acute pancreatitis
SD: Standard deviation
TDI: Townsend deprivation index
TyG: Triglyceride‐glucose
TyG‐BMI: TyG related to body mass index
TyG‐WC: TyG related to waist circumference
TyG‐WHtR: TyG related to waist‐to‐height ratio
VAI: Visceral adiposity index

## 1 Introduction

Acute pancreatitis (AP) is an inflammatory disease that originates from the pancreatic acinar cells. The main clinical symptom is a sudden continuous abdominal pain, accompanied by upregulated amylase and lipase in serum [1]. Most AP are mild and self‐limiting, requiring short‐term hospitalization. However, 20% of AP patients can develop a moderately severe or severe disease course, accompanied by complications of pancreatic necrosis or organ failure, leading to about a mortality rate of approximately 40% [2].

Alcohol abuse and gallstones are the most common causes of AP, other triggers include abnormal metabolism (e.g., hypertriglyceridemia), autoimmune, drug usage, infections, and trauma [3]. The global incidence of AP has been increasing at a rate of 2.30%–3.84% per year [4]. In the United States, almost 300,000 AP patients are admitted to the hospital each year, causing a cost of over 2.5 billion dollars [5]. Due to the increasing incidence and hospitalization of AP, there is an urgent need to establish effective and preventive interventions.

Insulin resistance (IR) is not only associated with an increased risk of AP but also serves as an independent prognostic factor [6]. Some detection methods are used for evaluating insulin sensitivity, such as the euglycemic insulin clamp, the hyperglycemic clamp, and insulin suppression [7]. However, these methods are either invasive, high‐cost, or time‐consuming, which hamper population screening and monitoring [8]. It is noteworthy that triglyceride‐related indices have been validated as appropriate surrogates for IR, such as the triglyceride‐glucose index (TyG), visceral adiposity index (VAI), lipid accumulation product (LAP), and atherogenic index of plasma (AIP) [9]. Studies showed that the TyG index is associated with a poor prognosis in AP patients [10], which is an effective biomarker for predicting severe acute pancreatitis (SAP) [11]. One study in north China reported that LAP and the cardiometabolic index (CMI), which were also considered surrogate indices of IR, were positively connected to the risk of AP [12]. Considering adiposity is associated with IR and AP [6], obesity‐associated parameters (body mass index, waist circumference, and height) were also taken into account in this analysis. Thus, we comprehensively investigated the association between triglyceride‐related indices and AP, aiming to find the most relevant index to AP and provide insight into AP risk assessment and management.

**TABLE 1:** Baseline characteristics of participants according to acute pancreatitis status at follow‐up.

Characteristics	Total (*n* = 387,275)	Non‐AP (*n* = 385,003)	Incident AP (*n* = 2272)	P value^a^
Age, years, mean (SD)	56.24 (8.10)	56.22 (8.10)	58.61 (7.76)	< 0.001
Sex, *n* (%)				< 0.001
Female	204,502 (52.8)	203,401 (52.8)	1101 (48.5)	
Male	182,773 (47.2)	181,602 (47.2)	1171 (51.5)	
Ethnic background, *n* (%)				0.011
White	365,940 (94.50)	363,765 (94.50)	2175 (95.70)	
Others	21,335 (5.50)	21,238 (5.50)	97 (4.30)	
Education level, *n* (%)				0.014
High school and below	342,831 (88.50)	340,782 (88.50)	2049 (90.20)	
College and above	44,444 (11.50)	44,221 (11.50)	223 (9.80)	
With college degree, *n* (%)				
TDI, mean (SD)	−1.30 (3.09)	−1.31 (3.09)	−0.80 (3.26)	< 0.001
BMI, kg/m^2^, mean (SD)	27.44 (4.78)	27.43 (4.77)	29.43 (5.45)	< 0.001
WC, cm, mean (SD)	90.42 (13.45)	90.39 (13.44)	96.24 (13.87)	< 0.001
Current drinker, *n* (%)	356,762 (92.10)	354,758 (92.10)	2004 (88.20)	< 0.001
Never smoker, *n* (%)	214,279 (55.30)	213,206 (55.40)	1073 (47.20)	< 0.001
Physical activity, *n* (%)				< 0.001
Low	58,782 (15.20)	58,342 (15.20)	440 (19.40)	
Moderate	127,608 (33.00)	126,912 (33.00)	696 (30.60)	
High	127,167 (32.80)	126,541 (32.90)	626 (27.60)	
Missing	73,718 (19.00)	73,208 (19.00)	510 (22.40)	
Diabetes, *n* (%)	19,843 (5.10)	19,635 (5.10)	208 (9.20)	< 0.001
Cholelithiasis, *n* (%)	6249 (1.60)	5992 (1.60)	257 (11.30)	< 0.001
Lipid‐lowering drugs, *n* (%)	66,459 (17.20)	65,873 (17.10)	586 (25.80)	< 0.001
TyG index, mean (SD)	8.71 (0.57)	8.71 (0.57)	8.89 (0.56)	< 0.001
TyG‐BMI index, mean (SD)	240.02 (49.71)	239.89 (49.65)	262.37 (54.68)	< 0.001
TyG‐WC index, mean (SD)	791.03 (147.76)	790.64 (147.67)	858.10 (149.10)	< 0.001
TyG‐WHtR index, mean (SD)	4.69 (0.83)	4.69 (0.83)	5.09 (0.85)	< 0.001
AIP index, mean (SD)	0.04 (0.30)	0.03 (0.30)	0.14 (0.29)	< 0.001
CMI index, mean (SD)	0.77 (0.68)	0.77 (0.68)	1.01 (0.84)	< 0.001
VAI index, mean (SD)	2.14 (1.68)	2.14 (1.67)	2.68 (2.00)	< 0.001
LAP index, mean (SD)	55.08 (47.15)	54.97 (47.08)	72.91 (55.05)	< 0.001

Abbreviations: AIP, atherogenic index of plasma; AP, acute pancreatitis; BMI, body mass index; CMI, cardiometabolic index; IPAQ, International Physical Activity Questionnaire; LAP, lipid accumulation product; SD, standard deviation; TDI, Townsend deprivation index; TyG, triglyceride‐glucose; TyG‐BMI, TyG related to body mass index; TyG‐WC, TyG related to waist circumference; TyG‐WHtR, TyG related to waist‐to‐height ratio; VAI, visceral adiposity index; WC, waist circumference.

^a^ P values were calculated by F‐tests (continuous variables) or Chi‐square tests (categorical variables).

## 2 Materials and Methods

### 2.1 Data Source and Study Design

UK Biobank, an ongoing population‐based prospective cohort study, served as the data source for this study. Almost 500,000 individuals, aged 40–69 years, were recruited from 22 assessment centers in England, Scotland, and Wales between 2006 and 2010 in the UK Biobank [13]. After recruitment, each participant signed an informed consent form, and a touchscreen questionnaire that covered information ranging from socioeconomic and demographic characteristics, lifestyles, medical information, environmental factors, and diet was completed by participants at baseline. Besides, genome‐wide genotyping and laboratory investigations were also performed.

**FIGURE 1:**
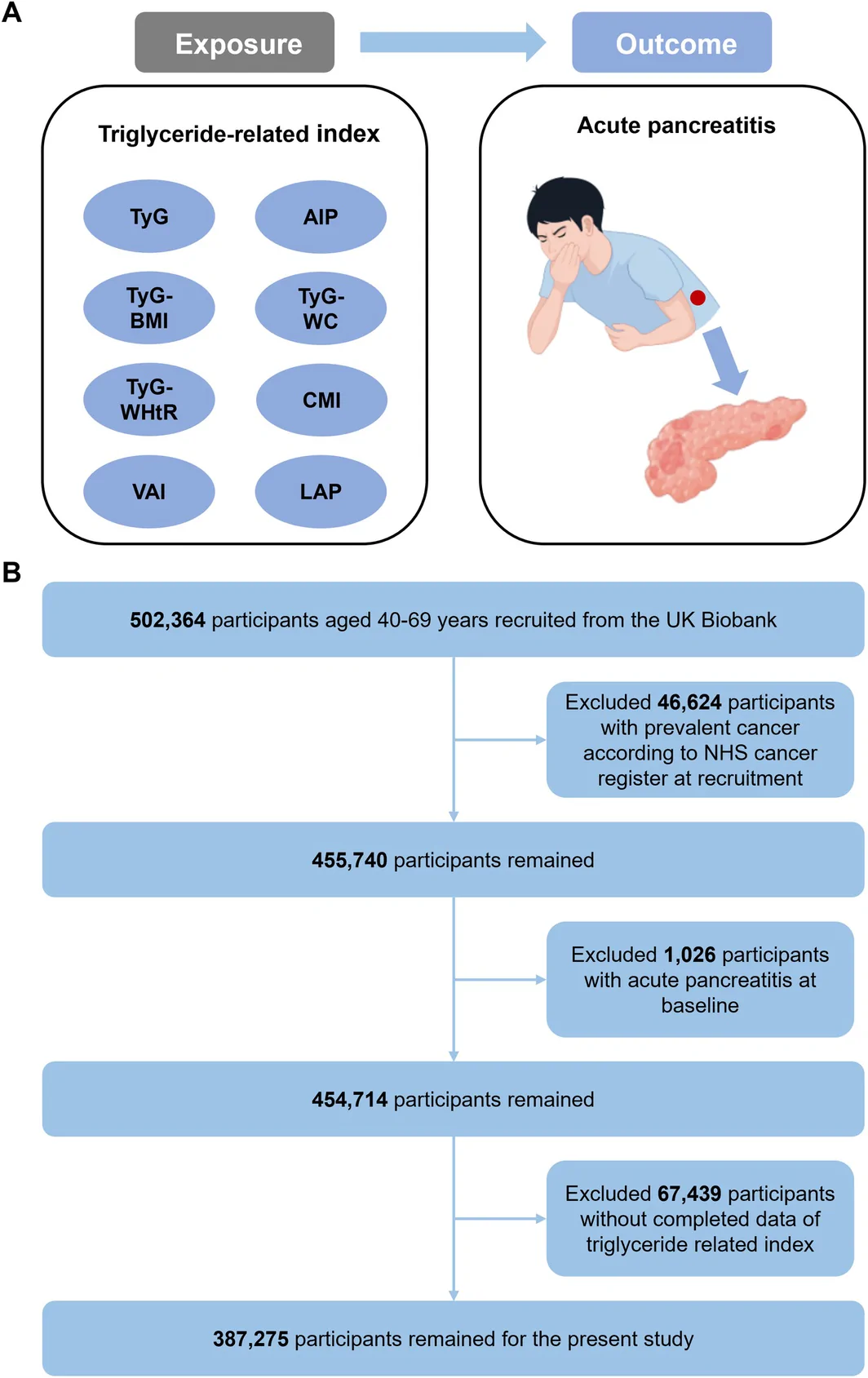
Study design and flowchart. (A) Study design; (B) Population screening.

In this study, among 502,364 participants who were recruited in the UK Biobank, those with cancer (*n* = 46,624) and AP (*n* = 1026) at baseline, as well as those with missing data on the triglyceride‐related indices (*n* = 67,439) were excluded. As a result, a total of 387,275 participants were finally included in this analysis (Figure 1).

### 2.2 Assessment of Triglyceride‐Related Indices

Peripheral venous blood samples were collected from all participants with an available sample at baseline, the detailed procedure for blood processing and biochemical analysis has been described elsewhere [14]. The specific formulas for triglyceride‐related indices are displayed in Table S2. Correlation between each triglyceride‐related index was calculated using Pearson's correlation coefficient. Meanwhile, we calculated the intraclass correlation coefficients (ICCs) and 95% confidence intervals (CIs) among triglyceride‐related indices between baseline measurements and repeated measurements to assess differential stability and change [15].

### 2.3 Assessment of Outcome

The outcome of interest was the incidence of AP during the follow‐up. The source of reported AP was ascertained from primary care records, hospital admissions data, death registers, and self‐reported diagnoses. Besides, AP was identified via the International Classification of Diseases 10 (ICD10) and ICD9 (Table S1). Participants were followed up from the date of recruitment until the date of AP event diagnosis, loss to follow‐up, or the end of follow‐up (December 19, 2022), whichever occurred first.

### 2.4 Assessment of Covariates

Covariates were incorporated based on scientific plausibility and prior literature [12], including age at recruitment, sex (male or female), ethnicity (white or others), education (college or below college), Townsend deprivation index (TDI), body mass index (BMI), smoking status (never or previous/current), alcohol consumption status (current or never/previous), physical activity (low, moderate or high), diabetes (yes or no), cholelithiasis (yes or no), and lipid‐lowering drug use (yes or no). Detailed covariate information is described in Table S1. The median values were imputed for missing continuous covariates, and the most populated category imputation was used for missing categorical variables.

### 2.5 Statistical Analysis

According to the AP status at follow‐up, we summarized and compared the baseline characteristics of individuals. For continuous variables with missing values, the median was used for imputation. Categorical variables with a missing rate of less than 3% were imputed using the maximum group. If a covariate had a missing rate of 3% or higher, the missing value was treated as a separate category. The categorical and continuous variables were presented as numbers (percentages) and means (standard deviations (SDs)). Differences between the Non‐AP and Incident AP groups were assessed using F‐tests and Chi‐square tests for continuous and categorical variables, respectively.

The multivariable Cox regression models were used to explore the associations between triglyceride‐related indices and the risk of AP. Model 1 was adjusted for age and sex; Model 2 was further adjusted for ethnicity, education, Townsend deprivation index, alcohol consumption status, smoking status, and physical activity; Model 3, based on Model 2, with adjustment of body mass index (only for TyG index and AIP index), diabetes, cholelithiasis, and lipid‐lowering drug. Based on these Cox models, we calculated the hazard ratios (HRs) and 95% CIs of triglyceride‐related indices in quartiles for incidents of AP. The lowest quartile was set as the reference group. A test for linear trend was assessed by assigning the median value of each exposure quartile. We also evaluated the HRs per SD increment in exposure variables.

To evaluate the dose–effect correlations between triglyceride‐related indices and AP, restricted cubic splines (RCS) were employed to evaluate and visualize the relationship using the rms R package. In addition, to assess heterogeneity, we conducted subgroup analyses for all covariates. P for interaction was calculated to assess the comparability in each subgroup. Three sets of sensitivity analyses were performed to verify the robustness of the results. First, to reduce potential reverse causation, we excluded incident AP events that occurred within the first two years of follow‐up and repeated the primary analyses. Second, to minimize biases owing to the imputation method, we performed primary analyses on participants without missing data. Third, we further adjusted CRP in Model 3, which may be a possible confounder, to test whether the association was weakened. An additional sensitivity analysis was conducted to evaluate the robustness to unmeasured confounders by using the E value methodology [16].

The receiver operating characteristic (ROC) curve analysis was used to evaluate the predictive power of triglyceride‐related indices at baseline for the risk of AP at follow‐up. Statistical tests were two‐sided, and P value < 0.05 was statistically significant. All statistical analyses were performed using R 4.3.3.

## 3 Results

### 3.1 Baseline Characteristics

Among 387,275 participants, 2272 participants with incident AP were recorded during a median follow‐up period of 13.70 years [interquartile range (IQR) 12.94–14.42]. The mean (SD) age was 56.24 (8.10) years and females accounted for 52.80%. The major ethnicity of the participants was white (94.50%). Compared to participants without AP, participants with incident AP were older and had a higher BMI, higher waist circumference (WC), but lower physical activity. Besides, participants with incident AP had higher levels of TyG, TyG‐BMI, TyG‐WC, TyG‐WHtR, AIP, CMI, VAI, and LAP index, and were more likely to have diabetes and cholelithiasis (Table 1). Figure S1 presents the Pearson's correlation coefficients between each triglyceride‐related index. CMI was most strongly correlated with VAI with a correlation coefficient of 0.96. Among the 13,299 participants who did not develop AP during the interval between the two measurements, high ICCs (all indices > 0.60) were found in triglyceride‐related indices between the initial and repeated assessments (Table S5).

### 3.2 Association of Triglyceride‐Related Indices With Incident AP

**FIGURE 2:**
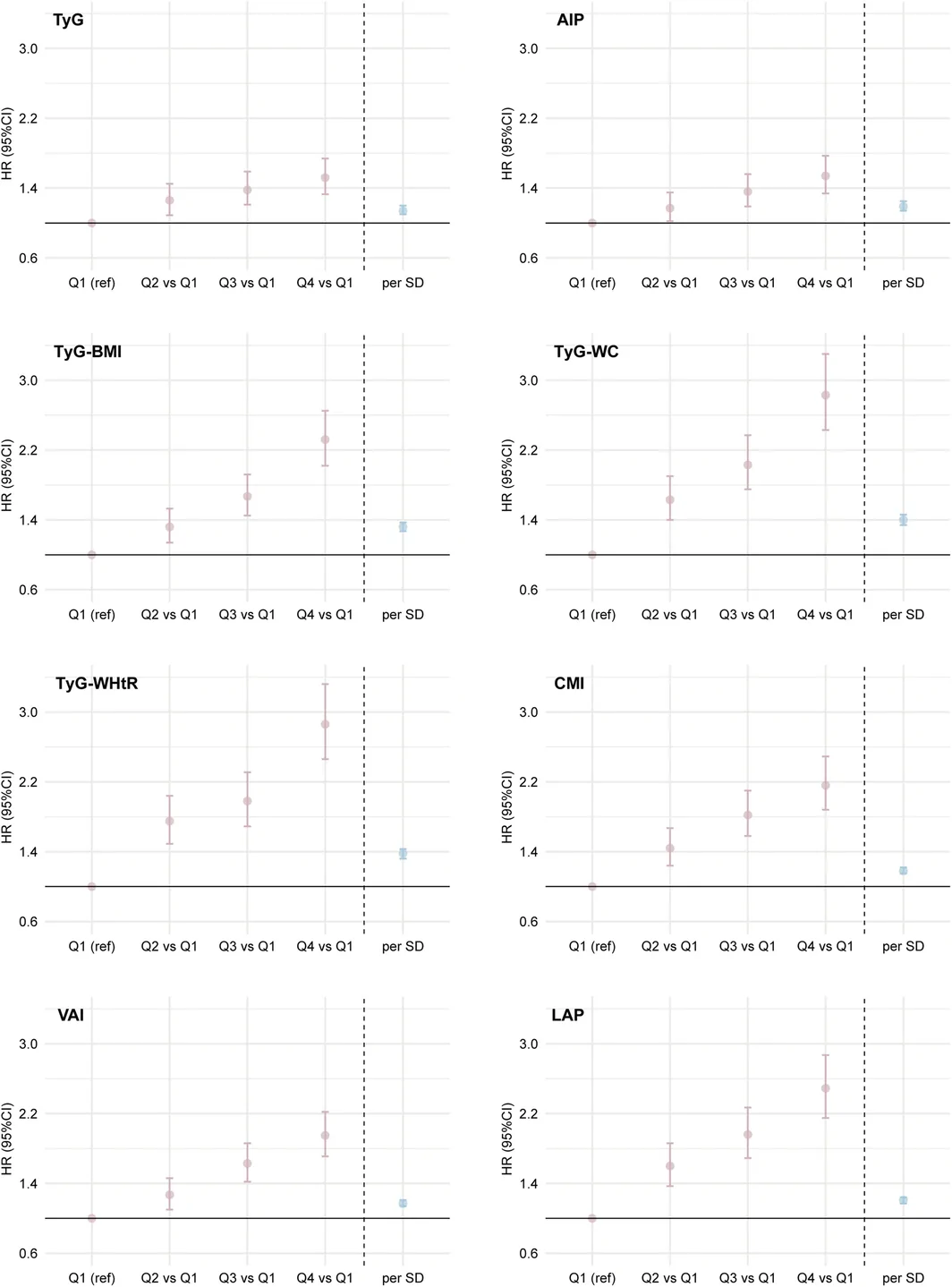
Multiple‐adjusted incident acute pancreatitis hazard ratios (HRs) with 95% confidence intervals (CIs) for triglyceride‐related index by quarters and per standard deviation.

After adjusting for all covariates mentioned above, the TyG‐WHtR had the highest association (HR 2.86, 95% CI 2.46–3.32), followed by TyG‐WC (HR 2.83, 95% CI 2.43–3.30) and LAP (HR 2.49, 95% CI 2.15–2.87). For the TyG index, when compared with the lowest quarter, the highest quarter had a higher risk of AP (HR 1.52, 95% CI 1.33–1.74) (P for trend < 0.001) (Figure 2; Table S4); when modeled as a continuous variable, the HR (95% CI) per SD was 1.14 (1.10, 1.20). For AIP index, the highest quarter was associated with a higher risk of AP when compared with the lowest quarter (HR 1.54, 95% CI 1.34–1.77) (P for trend < 0.001). A higher AIP was associated with a greater risk of AP (HR 1.19, 95% CI 1.14–1.25, per SD). For the TyG‐BMI, when compared with the lowest quarter, the risk of AP associated with the highest quarter was higher (HR 2.32, 95% CI 2.02–2.65) (P for trend < 0.001), and the HR per SD was 1.32 (1.27–1.37). The highest quarter of CMI was associated with a higher risk of AP when compared with the lowest quarter (HR 2.16, 95% CI 1.88–2.49). A higher CMI was associated with an increased risk of AP (HR 1.18, 95% CI 1.15–1.22, per SD). For VAI, when compared with the lowest quarter, the highest quarter was associated with a greater risk of AP (HR 1.95, 95% CI 1.71–2.22) (P for trend < 0.001), and a higher value indicated a greater risk of AP (HR 1.17, 95% CI 1.14–1.21, per SD).

**FIGURE 3:**
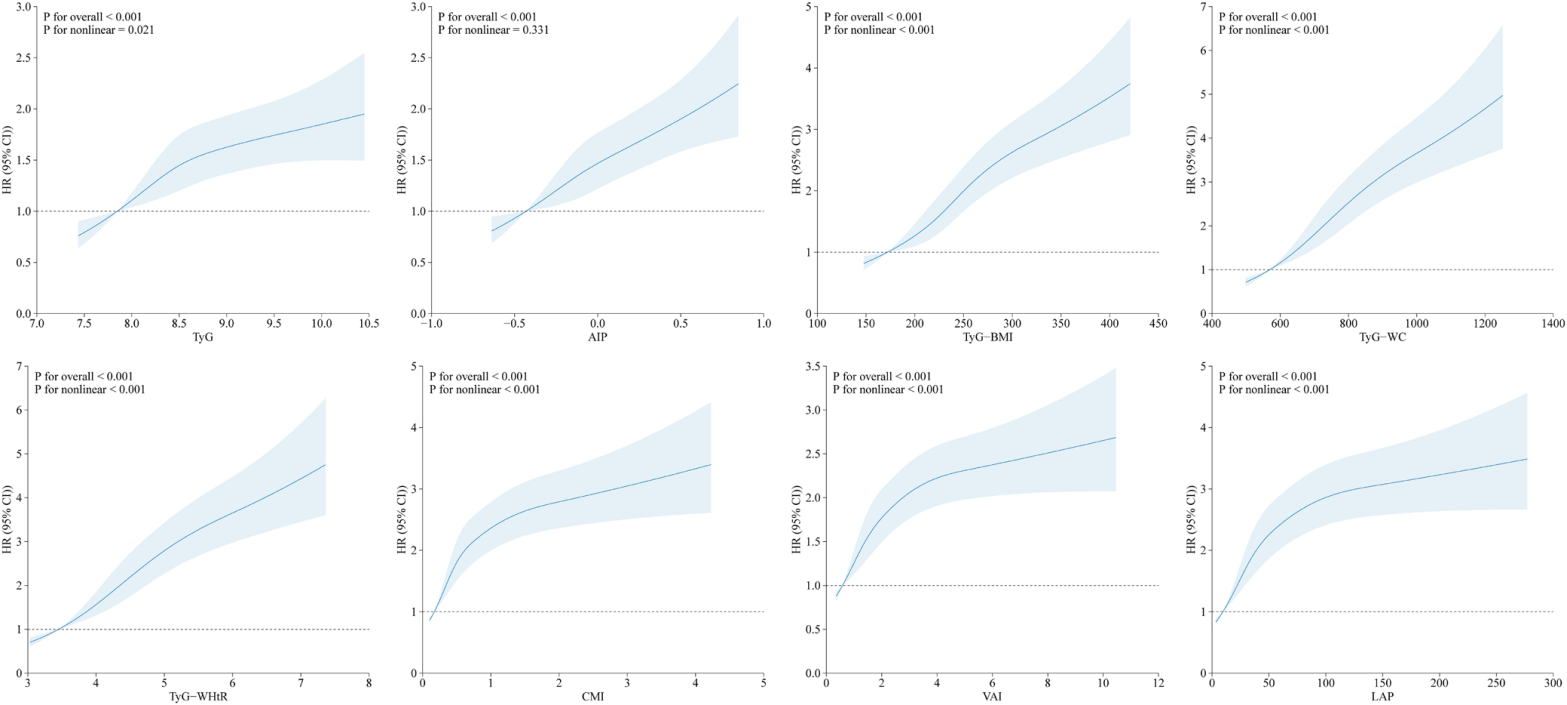
The restricted cubic spline (RCS) analysis between the triglyceride‐related index and the risk of acute pancreatitis.

Possible nonlinear relationships (P for nonlinear < 0.05) between triglyceride‐related indices, except for the AIP index, were examined with multivariable‐adjusted RCS analyses (Figure 3).

### 3.3 The Predictive Value of Each Index for AP

**FIGURE 4:**
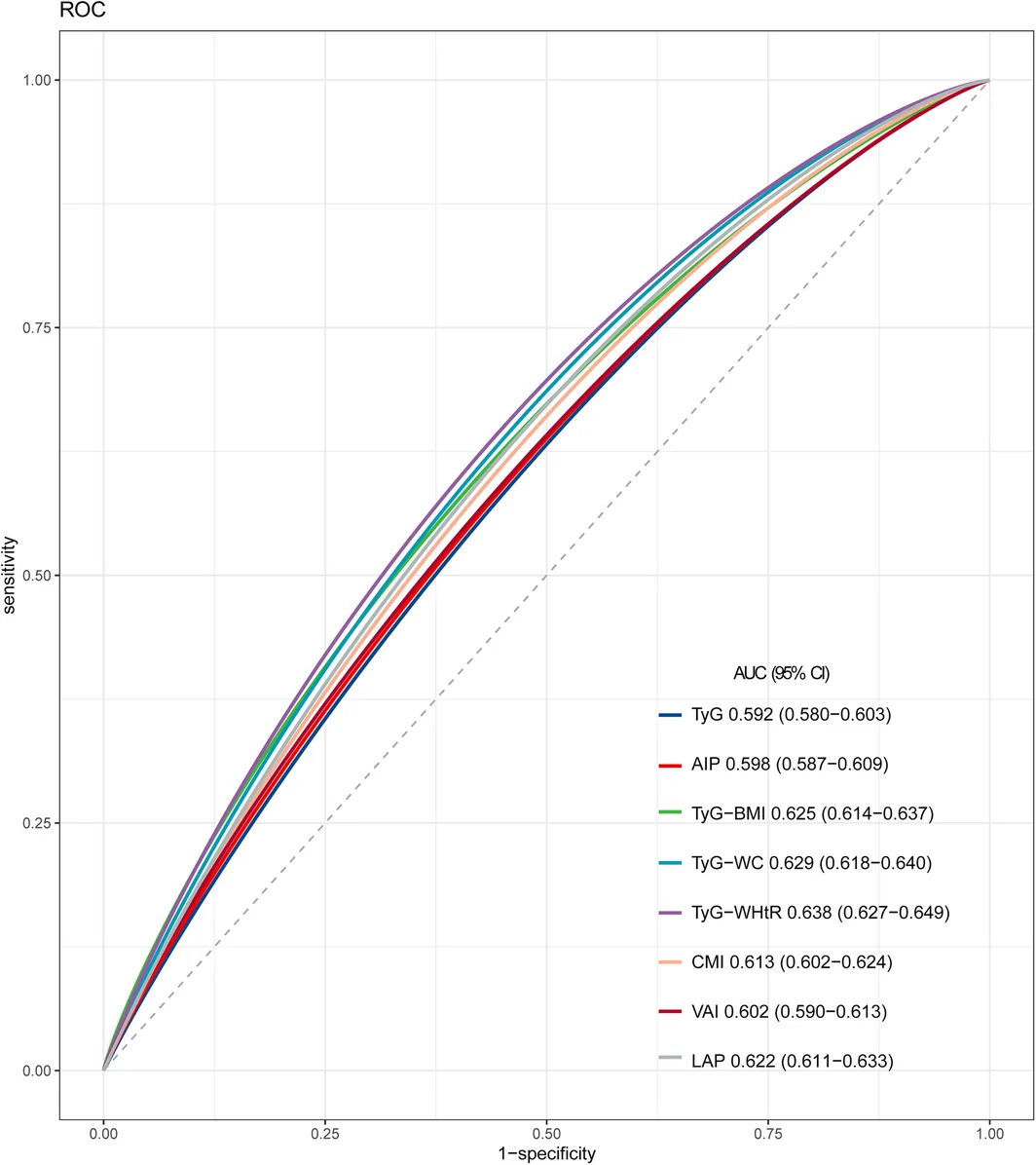
ROC curve for each index as predictors of incident acute pancreatitis.

Figure 4 shows the ROC curve of different indices. The area under the curve (AUC) was used to evaluate the performance of predictions. The TyG‐WHtR index had the highest AUC at 0.638 (95% CI: 0.627–0.649), closely followed by the TyG‐WC index with an AUC of 0.629 (95% CI: 0.618–0.640) and the TyG‐BMI index at 0.625 (95% CI: 0.614–0.637). The TyG index recorded a lower AUC of 0.592 (95% CI: 0.580–0.603), while the AIP index had an AUC of 0.598 (95% CI: 0.587–0.609).

### 3.4 Subgroup Analysis and Sensitivity Analysis

To elucidate whether the effect of the triglyceride‐related indices differed among subgroups of covariates, we performed covariate‐based subgroup analysis between each triglyceride‐related index (Figure S2‐S9). For TyG index, significant interactions were observed in the sex subgroup, BMI subgroup, diabetes subgroup, and cholelithiasis subgroup (P for interaction < 0.05). Specifically, the association between TyG and AP was statistically significant among participants without cholelithiasis or BMI larger than 25. Additionally, subgroup analysis according to alcohol consumption did not show interactions between subgroups (P for interaction = 0.124) (Figure S2). There were no significant interactions between AIP and the subgroup factors except for education, TDI, and BMI (Figure S3). When subgroups were carried out for the TyG‐BMI index, significant interactions were revealed in TDI, alcohol consumption, smoking, diabetes, and cholelithiasis subgroups (P for interaction < 0.05). Specifically, the relationship between TyG‐BMI and onset AP was not significant among participants with diabetes or cholelithiasis (Figure S4). The relationship patterns were similar when exploring the TyG‐WHtR index (Figure S6). For the TyG‐WC index, the interaction analysis indicated a nonsignificant interaction effect across all subgroups except for the BMI, physical activity, and cholelithiasis subgroup (Figure S5). Besides, no statistically significant interactions between CMI and these subgroup characteristics, including alcohol consumption, BMI, diabetes, and cholelithiasis (all P for interaction > 0.05) (Figure S7). For the VAI index, TDI, BMI, physical activity, and diabetes subgroup displayed significant interactions (Figure S8). Moreover, there were no significant interactions between LAP and the subgroup factors except for TDI, physical, and cholelithiasis (Figure S9), and the association between LAP and AP remained significant among participants without cholelithiasis.

In sensitivity analysis, after excluding participants diagnosed with AP within the first two years, the results were consistent with the primary findings (Table S6). Similarly, the results were still robust in the study population without missing data (Table S7). We also found similar results with additional adjustment for CRP (Table S8).

## 4 Discussion

In this large population‐based prospective cohort study, we found associations between triglyceride‐related indices and the risk of AP: elevated baseline TyG, AIP, TyG‐BMI, TyG‐WHtR, TyG‐WC, CMI, VAI, and LAP index were associated with a higher risk of AP occurrence. We found nonlinear relationships between these triglyceride‐related indices except for AIP and the risk of AP. In addition, all indices exhibited the capability to predict the risk of AP (all AUC > 0.5), and the TyG‐WHtR was superior to other indices for predicting AP.

IR is a hallmark of diabetes, which is an important factor linked to the susceptibility of diabetes and AP [17]. IR can promote the development and progression of AP through dysregulating lipid metabolism and chronic inflammation, thus exacerbating pancreatic stress [18, 19]. Regrettably, the methods to assess IR are sophisticated and unsuitable for large‐scale screening. The TyG index, a surrogate for IR, is commonly used to assess metabolic diseases [20]. Previous studies invariably concentrated on the relationship between TyG index and the severity of AP, which indicated that TyG index was an independent risk factor for SAP [10]. Our research first demonstrated a positive association between the TyG index and the risk of incident AP. Obesity not only increased AP incidence but also worsened AP severity [21]. Especially, central obesity is an important risk factor for IR [22]. Several studies indicated that obesity‐related metrics, like some simple anthropometric parameters, combined with TyG were closely associated with IR [23, 24]. One single‐center observational study reported that a higher TyG‐BMI was positively correlated with increased all‐cause mortality of AP patients [25]. Our study prospectively found TyG‐BMI, TyG‐WC, and TyG‐WHtR were associated with a higher risk of AP. AIP index, the combination of TG and HDL‐C, correlating with dysfunctional glucose metabolism, reflects the severity of IR [26]. It is also considered an indicator of coronary syndrome and metabolic syndrome [27, 28]. A cross‐sectional study demonstrated that AIP was significantly associated with SAP [29]. While in this study, we found for the first time that higher AIP is a risk factor for onset AP. One prospective study conducted in China found CMI and LAP were positively connected to the risk of AP [12]. The results were consistent with our findings. Some studies reported CMI was associated with obesity‐associated metabolic disorders such as cardiovascular diseases and diabetes [30, 31]. LAP, a new anthropometric measure of lipid over accumulation, which combines TG and WC, is used as an indicator of visceral adipose tissue and abdominal lipid accumulation [32]. It is found that LAP is a robust marker for predicting diabetes, metabolic syndrome, IR, and nonalcoholic fatty liver disorder (NAFLD) [33, 34]. As with LAP, VAI is a reliable indicator evaluating the distribution and dysfunction of visceral fat [35]. This research found that VAI is positively associated with the occurrence of AP. Meanwhile, one retrospective study reported that VAI was positively correlated with the severity of hyperlipidemic AP [36], which indicated that VAI was closely related to the occurrence and development of AP.

Previous studies indicated that the TyG index was higher in the SAP group compared to the non‐SAP group, which was considered an independent predictive factor for SAP [10, 37]. One retrospective cohort study found that compared with mild AP, SAP patients had higher levels of VAI, LAP, and CMI. Among these indices, VAI displayed the strongest predictive ability of SAP [38]. Meanwhile, it was reported that AIP was a superior predictive factor to TG or HDL cholesterol level alone for predicting the risk of SAP [29]. All previous studies focused on the predictive ability of the severity of AP by triglyceride‐related indices, but data on the occurrence of AP were lacking. Our study filled this gap and found that triglyceride‐related indices had the ability to predict the onset of AP. The ROC curve displayed that the predictive efficacy of TyG‐BMI, TyG‐WC, and TyG‐WHtR with AP was, to some extent, superior to the TyG index. Among these indices, TyG‐WHtR had the best predictive efficacy. This superior performance may be attributed to its integration of the TyG index and WHtR, the latter of which is recognized as a robust marker of central adiposity [39]. Central adiposity is a key driver of systemic inflammation and the release of pro‐inflammatory cytokines, potentially contributing to the pathogenesis and development of AP [38].

To our knowledge, this is the first large‐scale prospective cohort study comprehensively investigating the association between triglyceride‐related indices and the risk of AP. However, several limitations existed in our study. First, despite adjustments for some major confounders, residual confounding effects cannot be excluded. We calculated E‐values to assess the potential impact of unmeasured confounders; the relatively large E‐values indicated unmeasured potential confounders could not overcome the effect of triglyceride‐related indices observed in the current analysis (Table S4). Second, it is worth noting that while homeostatic model assessment for insulin resistance (HOMA‐IR) is a widely recognized metric for IR, fasting insulin levels were not available in the UK Biobank database, which precluded its calculation in the present study. Instead, we utilized triglyceride‐related indices (such as TyG, TyG‐BMI, TyG‐WC, and TyG‐WHtR), which are validated surrogates for IR in large‐scale studies [40]. Our analysis confirms these surrogates are significantly associated with AP risk and offer effective predictive value. Lastly, since the determination of triglyceride‐related indices was based on baseline blood samples and anthropometric measurements, the changes in these indices or the effects of any change could not be fully assessed during the observed period. The high ICCs in a subsample with repeated assessments suggested that time‐dependent variation in triglyceride‐related indices was unlikely to have a significant impact on our results.

## 5 Conclusion

Elevated triglyceride‐related indices were related to heightened AP risk. Among these indices, the TyG‐WHtR index had the strongest predictive power on the onset of AP. Our study suggested prevention of IR may reduce the risk of AP. Assessments of triglyceride‐related indices may be beneficial for identifying people at early risk of AP and reducing the cost of screening.

## Funding

This work was supported by the Science and Technology Innovation Program of Hunan Province (2022RC1020), the Wisdom Accumulation and Talent Cultivation Project of the Third Xiangya Hospital of Central South University (YX202208), and the National Natural Science Foundation of China (82472736).

## Ethics Statement

This research has been conducted using the UK Biobank Resource under Application Number 103631, and the UK Biobank obtained ethical approval from the National Health Service (NHS) North West Multi‐Center Research Ethics Committee (protocol code 11/NW/0382). This work uses data provided by patients and collected by the NHS as part of their care and support. The linked health data acknowledgement can be found at https://www.ukbiobank.ac.uk/enable‐your‐research/manage‐your‐project.

## Conflicts of Interest

The authors declare no conflicts of interest.

## Supporting information


**Data S1:** kjm270189‐sup‐0001‐Supinfo.docx.

## Data Availability

The data that support the findings of this study are available from the UK Biobank (www.ukbiobank.ac.uk), but restrictions apply to the availability of these data, which were used under Application Number 103631 for the current study, and are not publicly available. Data are however available from the authors upon reasonable request and with the permission of the UK Biobank.
