# Supplementary material for: Triglyceride‐Related Indices and Risk of Acute Pancreatitis: A Prospective Study in UK Biobank
Source: Kaohsiung J Med Sci. 2026 Mar 10;42(9):e70189. doi: 10.1002/kjm2.70189 (PMC13399814; doi:10.1002/kjm2.70189)
Supplement: Supplementary file 1 [file KJM2-42-e70189-s001.docx]

**Supplementary Materials**

**Triglyceride-related indices and risk of acute pancreatitis: a prospective study in UK Biobank**

[Supplementary Tables	3](#_Toc218965254)

[Table S1. Diagnostic, description, and classification of acute pancreatitis and covariates.	3](#_Toc218965255)

[Table S2. Calculation of triglyceride related-indices.	6](#_Toc218965256)

[Table S3. The numbers and percentages of missing data of covariates.	7](#_Toc218965257)

[Table S4. The association of triglyceride-related index with the risk of acute pancreatitis.	8](#_Toc218965258)

[Table S5. Intraclass correlation coefficient between two repeated measurements of triglyceride-related indices.	11](#_Toc218965259)

[Table S6. Sensitivity analysis shows the robust association between the triglyceride-related indices with the risk of acute pancreatitis (excluding participants diagnosed with acute pancreatitis within the first two years after baseline).	12](#_Toc218965260)

[Table S7. Sensitivity analysis shows the robust association between the triglyceride-related indices with the risk of acute pancreatitis (excluding participants with missing covariate data).	15](#_Toc218965261)

[Table S8. Sensitivity analysis shows the robust association between the triglyceride-related indices with the risk of acute pancreatitis (further adjusted for c-reactive protein).	18](#_Toc218965262)

[Supplementary Figures	21](#_Toc218965263)

[Figure S1. Correlation matrix for triglyceride-related indices, using Pearson’s correlation.	21](#_Toc218965264)

[Figure S2. Subgroup analysis of TyG index and acute pancreatitis.	22](#_Toc218965265)

[Figure S3. Subgroup analysis of AIP index and acute pancreatitis.	23](#_Toc218965266)

[Figure S4. Subgroup analysis of TyG-BMI index and acute pancreatitis.	24](#_Toc218965267)

[Figure S5. Subgroup analysis of TyG-WC index and acute pancreatitis.	25](#_Toc218965268)

[Figure S6. Subgroup analysis of TyG-WHtR index and acute pancreatitis.	26](#_Toc218965269)

[Figure S7. Subgroup analysis of CMI index and acute pancreatitis.	27](#_Toc218965270)

[Figure S8. Subgroup analysis of VAI index and acute pancreatitis.	28](#_Toc218965271)

[Figure S9. Subgroup analysis of LAP index and acute pancreatitis.	29](#_Toc218965272)

# Supplementary Tables

# Table S1. Diagnostic, description, and classification of acute pancreatitis and covariates.

| **Variable** | **Diagnostic codes/ Description** |
| --- | --- |
| **Acute pancreatitis** | **ICD 10 codes:**  K85 Acute pancreatitis  K85.0 Idiopathic acute pancreatitis  K85.1 Biliary acute pancreatitis  K85.2 Alcohol-induced acute pancreatitis  K85.3 Drug-induced acute pancreatitis  K85.8 Other acute pancreatitis  K85.9 Acute pancreatitis, unspecified  **ICD 9 codes:**  5770 Acute pancreatitis |
| **Cholelithiasis** | **ICD 10 codes:**  K80 Cholelithiasis  K80.0 Calculus of gallbladder with acute cholecystitis  K80.1 Calculus of gallbladder with other cholecystitis  K80.2 Calculus of gallbladder without cholecystitis  K80.3 Calculus of bile duct with cholangitis  K80.4 Calculus of bile duct with cholecystitis  K80.5 Calculus of bile duct without cholangitis or cholecystitis  K80.8 Other cholelithiasis  **ICD 9 codes:**  574 Cholelithiasis  5740 Calculus of gallbladder with acute cholecystitis  5741 Calculus of gallbladder with other cholecystitis  5742 Calculus of gallbladder without mention of cholecystitis  5743 Calculus of bile duct with acute cholecystitis  5744 Calculus of bile duct with other cholecystitis  5745 Calculus of bile duct without mention of cholecystitis |
| **Glucose** | Measured by hexokinase analysis on a Beckman Coulter AU5800, units of measurement are mmol/L. |
| **Triglycerides** | Measured by GPO-POD analysis on a Beckman Coulter AU5800, units of measurement are mmol/L. |
| **HDL cholesterol** | Measured by enzyme immunoinhibition analysis on a Beckman Coulter AU5800, units of measurement are mmol/L. |
| **C-reactive protein** | Measured by immunoturbidimetric - high sensitivity analysis on a Beckman Coulter AU5800, units of measurement are mg/L. |
| **Sex** | Sex was dichotomized into female or male. |
| **Ethnicity** | The detailed information of ethnic background is available at UK Biobank Data-Field 21000. We treated it as a binary variable, white (including British, Irish, any other White background) or others (all options except British, Irish, any other White background). |
| **Townsend deprivation index** | Townsend deprivation index was quantified based on home ownership, employment, household overcrowding, and car ownership ^(1)^. Townsend deprivation index calculated immediately prior to participant joining UK Biobank. Each participant is assigned a score corresponding to the output area in which their postcode is located (Based on the preceding national census output areas). |
| **Education level** | ACE touchscreen question "Which of the following qualifications do you have? (You can select more than one)". The educational qualification categories were as follows: “College or University degree,” “A levels/AS levels or equivalent,” “O levels/GCSEs or equivalent,” “CSEs or equivalent,” “NVQ or HND or HNC or equivalent,” “Other professional qualifications, e.g., nursing, teaching,” “None of the above,” and “Prefer not to answer.” This was treated as a binary variable, college (College or University degree) or below college (except for the first and last options). |
| **Weight** | Weight was measured by a variety of means during the initial Assessment Centre visit, units of measurement are kg. |
| **Standing height** | Standing height was measured using a Seca 202 device, units of measurement are cm. |
| **Body mass index** | BMI value is constructed from height and weight measured during the initial Assessment Centre visit, units of measurement are kg/m^2^. |
| **Waist circumference** | Units of measurement are cm. |
| **Physical activity** | Participants were asked questions from the International Physical Activity Questionnaire form to evaluate their daily activities at baseline. Participants were divided into three categories: low, moderate, and high physical activity. |
| **Alcohol drinker status** | The alcohol drinker status available for participants to choose from are: “Never”, “Previous”, “Current”, “Prefer not to answer”. We treated it as a binary variable, current drinker (current) and not current drinker (never and previous). |
| **Smoking status** | The smoking status available for participants to choose from are the same as alcohol drinker status. We treated it as a binary variable, never smoker (never) and not never smoker (previous and current). |
| **Diabetes** | ACE touchscreen question "Has a doctor ever told you that you have diabetes?". It was treated as a binary variable (“Yes” or “No”) |
| **Lipid-lowering drug** | ACE touchscreen question "Do you regularly take any of the following medications? (You can select more than one answer)". If participants chose the “Cholesterol lowering medication” option, we defined them as “Yes”; otherwise, “No”. |

# Table S2. Calculation of triglyceride related-indices.

| **Index** | **Formula** |
| --- | --- |
| TyG ^(2)^ | ln (FBG (mg/dL) × TG (mg/dL)/2) |
| TyG-BMI ^(3)^ | TyG × BMI |
| TyG-WC ^(3)^ | TyG × WC (cm) |
| TyG-WHtR^(4)^ | TyG × WC (cm)/ height (cm) |
| AIP ^(5)^ | log10 (TG (mg/dL)/ HDL-C (mg/dL)) |
| CMI ^(6)^ | TG (mmol/L)/ HDL-C (mmol/L) × WC (cm)/ height (cm) |
| VAI ^(7)^ | Male: {WC (cm)/ (39.68 + (1.88 × BMI))} × (TG (mmol/L)/ 1.03) × (1.31/ HDL-C (mmol/L))  Female: {WC (cm)/ (36.58 + (1.89 × BMI))} × (TG (mmol/L)/ 0.81) × (1.52/ HDL-C (mmol/L)) |
| LAP ^(8)^ | Male: (WC (cm) − 65) × TG (mmol/L)  Female: (WC (cm) − 58) × TG (mmol/L) |

Abbreviations: FBG, fasting blood glucose; TG, triglyceride; TyG, triglyceride-glucose; BMI, body mass index; WC, waist circumference; HDL-C, high-density lipoprotein cholesterol; AIP, atherogenic index of plasma; CMI, cardiometabolic index; VAI, visceral adiposity index; LAP, lipid accumulation product.

# Table S3. The numbers and percentages of missing data of covariates.

| **Covariates** | **Missing number** | **Missing rate (%)** |
| --- | --- | --- |
| Age | 0 | 0 |
| Sex | 0 | 0 |
| Ethnicity | 1,832 | 0.47 |
| Education | 4,170 | 1.08 |
| Townsend deprivation index | 469 | 0.12 |
| Body mass index | 0 | 0 |
| Waist circumference | 0 | 0 |
| Alcohol status | 0 | 0 |
| Smoking status | 955 | 0.25 |
| Physical activity | 1,908 | 0.49 |
| Diabetes | 73,718 | 19.04 |
| Cholelithiasis | 1,685 | 0.44 |
| Lipid-lowering drugs | 0 | 0 |

# Table S4. The association of triglyceride-related index with the risk of acute pancreatitis.

|  | **Events (n)** | **Person-years** | **Model 0** | | **Model 1** | | **Model 2** | | **Model 3** | | |
| --- | --- | --- | --- | --- | --- | --- | --- | --- | --- | --- | --- |
|  |  |  | **HR (95% CI)** | **P value** | **HR (95% CI)** | **P value** | **HR (95% CI)** | **P value** | **HR (95% CI)** | **P value** | **E-value (CI)** |
| **TyG** |  |  |  |  |  |  |  |  |  |  |  |
| Q1 (5.86 to ≤ 8.31) | 327 | 1,305,945 | Reference |  | Reference |  | Reference |  | Reference |  | Reference |
| Q2 (8.31 to ≤ 8.68) | 505 | 1,295,034 | 1.56 (1.36, 1.79) | <0.001 | 1.42 (1.23, 1.63) | <0.001 | 1.39 (1.21, 1.60) | <0.001 | 1.26 (1.09, 1.45) | 0.001 | 1.83 (1.40) |
| Q3 (8.68 to ≤ 9.08) | 640 | 1,290,074 | 1.98 (1.74, 2.27) | <0.001 | 1.75 (1.53, 2.00) | <0.001 | 1.67 (1.46, 1.91) | <0.001 | 1.38 (1.21, 1.59) | <0.001 | 2.10 (1.71) |
| Q4 (9.08 to ≤ 12.46) | 800 | 1,282,355 | 2.50 (2.19, 2.84) | <0.001 | 2.19 (1.92, 2.50) | <0.001 | 2.02 (1.77, 2.30) | <0.001 | 1.52 (1.33, 1.74) | <0.001 | 2.41 (1.99) |
| P for trend ^a^ | |  |  | <0.001 |  | <0.001 |  | <0.001 |  | <0.001 |  |
| Per SD increase | |  | 1.36 (1.31, 1.41) | <0.001 | 1.31 (1.26, 1.37) | <0.001 | 1.27 (1.22, 1.32) | <0.001 | 1.14 (1.10, 1.20) | <0.001 | 1.54 (1.43) |
| **AIP** |  |  |  |  |  |  |  |  |  |  |  |
| Q1 (-1.03 to ≤ -0.18) | 332 | 1,301,512 | Reference |  | Reference |  | Reference |  | Reference |  | Reference |
| Q2 (-0.18 to ≤ 0.03) | 478 | 1,295,849 | 1.45 (1.26, 1.66) | <0.001 | 1.37 (1.19, 1.58) | <0.001 | 1.33 (1.15, 1.53) | <0.001 | 1.17 (1.02, 1.35) | 0.029 | 1.62 (1.16) |
| Q3 (0.03 to ≤ 0.24) | 647 | 1,290,113 | 1.97 (1.72, 2.25) | <0.001 | 1.82 (1.59, 2.09) | <0.001 | 1.71 (1.50, 1.96) | <0.001 | 1.36 (1.19, 1.56) | <0.001 | 2.06 (1.67) |
| Q4 (0.24 to ≤ 1.33) | 815 | 1,285,934 | 2.49 (2.19, 2.83) | <0.001 | 2.35 (2.06, 2.68) | <0.001 | 2.12 (1.85, 2.42) | <0.001 | 1.54 (1.34, 1.77) | <0.001 | 2.45 (2.01) |
| P for trend | |  |  | <0.001 |  | <0.001 |  | <0.001 |  | <0.001 |  |
| Per SD increase | |  | 1.40 (1.34, 1.45) | <0.001 | 1.39 (1.33, 1.45) | <0.001 | 1.34 (1.28, 1.39) | <0.001 | 1.19 (1.14, 1.25) | <0.001 | 1.67 (1.54) |
| **TyG-BMI** |  |  |  |  |  |  |  |  |  |  |  |
| Q1 (96.03 to ≤ 204.76) | 293 | 1,303,621 | Reference |  | Reference |  | Reference |  | Reference |  | Reference |
| Q2 (204.76 to ≤ 233.48) | 442 | 1,297,241 | 1.52 (1.31, 1.76) | <0.001 | 1.39 (1.20, 1.61) | <0.001 | 1.38 (1.19, 1.60) | <0.001 | 1.32 (1.14, 1.53) | <0.001 | 1.97 (1.54) |
| Q3 (233.48 to ≤ 267.54) | 605 | 1,292,178 | 2.09 (1.81, 2.40) | <0.001 | 1.87 (1.62, 2.15) | <0.001 | 1.80 (1.56, 2.07) | <0.001 | 1.67 (1.45, 1.92) | <0.001 | 2.73 (2.26) |
| Q4 (267.54 to ≤ 715.96) | 932 | 1,280,367 | 3.25 (2.85, 3.70) | <0.001 | 2.96 (2.59, 3.37) | <0.001 | 2.66 (2.32, 3.04) | <0.001 | 2.32 (2.02, 2.65) | <0.001 | 4.07 (3.46) |
| P for trend | |  |  | <0.001 |  | <0.001 |  | <0.001 |  | <0.001 |  |
| Per SD increase | |  | 1.46 (1.41, 1.51) | <0.001 | 1.45 (1.40, 1.50) | <0.001 | 1.39 (1.34, 1.44) | <0.001 | 1.32 (1.27, 1.37) | <0.001 | 1.97 (1.86) |
| **TyG-WC** |  |  |  |  |  |  |  |  |  |  |  |
| Q1 (151.65 to ≤ 681.59) | 258 | 1,310,728 | Reference |  | Reference |  | Reference |  | Reference |  | Reference |
| Q2 (681.59 to ≤ 782.92) | 467 | 1,299,570 | 1.83 (1.57, 2.13) | <0.001 | 1.78 (1.53, 2.08) | <0.001 | 1.73 (1.48, 2.02) | <0.001 | 1.63 (1.40, 1.90) | <0.001 | 2.64 (2.15) |
| Q3 (782.92 to ≤ 886.11) | 614 | 1,290,777 | 2.42 (2.09, 2.80) | <0.001 | 2.40 (2.07, 2.80) | <0.001 | 2.26 (1.94, 2.63) | <0.001 | 2.03 (1.75, 2.37) | <0.001 | 3.48 (2.90) |
| Q4 (886.11 to ≤ 1875.74) | 933 | 1,272,333 | 3.74 (3.26, 4.29) | <0.001 | 3.79 (3.27, 4.39) | <0.001 | 3.33 (2.87, 3.87) | <0.001 | 2.83 (2.43, 3.30) | <0.001 | 5.11 (4.29) |
| P for trend | |  |  | <0.001 |  | <0.001 |  | <0.001 |  | <0.001 |  |
| Per SD increase | |  | 1.53 (1.47, 1.59) | <0.001 | 1.55 (1.49, 1.61) | <0.001 | 1.47 (1.41, 1.53) | <0.001 | 1.40 (1.34, 1.46) | <0.001 | 2.15 (2.01) |
| **TyG-WHtR** |  |  |  |  |  |  |  |  |  |  |  |
| Q1 (0.83 to ≤ 4.08) | 238 | 1,309,583 | Reference |  | Reference |  | Reference |  | Reference |  | Reference |
| Q2 (4.08 to ≤ 4.63) | 477 | 1,299,503 | 2.02 (1.73, 2.36) | <0.001 | 1.87 (1.60, 2.19) | <0.001 | 1.83 (1.57, 2.14) | <0.001 | 1.75 (1.49, 2.04) | <0.001 | 2.90 (2.34) |
| Q3 (4.63 to ≤ 5.20) | 590 | 1,290,823 | 2.52 (2.17, 2.93) | <0.001 | 2.26 (1.94, 2.64) | <0.001 | 2.15 (1.84, 2.51) | <0.001 | 1.98 (1.69, 2.31) | <0.001 | 3.37 (2.77) |
| Q4 (5.20 to ≤ 10.84) | 967 | 1,273,498 | 4.19 (3.64, 4.83) | <0.001 | 3.72 (3.21, 4.30) | <0.001 | 3.30 (2.85, 3.83) | <0.001 | 2.86 (2.46, 3.32) | <0.001 | 5.17 (4.36) |
| P for trend | |  |  | <0.001 |  | <0.001 |  | <0.001 |  | <0.001 |  |
| Per SD increase | |  | 1.56 (1.50, 1.62) | <0.001 | 1.52 (1.46, 1.58) | <0.001 | 1.44 (1.39, 1.50) | <0.001 | 1.38 (1.32, 1.43) | <0.001 | 2.10 (1.97) |
| **CMI** |  |  |  |  |  |  |  |  |  |  |  |
| Q1 (0.02 to ≤ 0.33) | 292 | 1,303,918 | Reference |  | Reference |  | Reference |  | Reference |  | Reference |
| Q2 (0.33 to ≤ 0.57) | 477 | 1,296,478 | 1.64 (1.42, 1.90) | <0.001 | 1.55 (1.34, 1.79) | <0.001 | 1.50 (1.30, 1.74) | <0.001 | 1.44 (1.24, 1.67) | <0.001 | 2.24 (1.79) |
| Q3 (0.57 to ≤ 0.98) | 660 | 1,289,491 | 2.29 (1.99, 2.63) | <0.001 | 2.12 (1.84, 2.43) | <0.001 | 1.98 (1.72, 2.28) | <0.001 | 1.82 (1.58, 2.10) | <0.001 | 3.04 (2.54) |
| Q4 (0.98 to ≤ 15.91) | 843 | 1,283,520 | 2.94 (2.57, 3.36) | <0.001 | 2.77 (2.41, 3.18) | <0.001 | 2.47 (2.15, 2.84) | <0.001 | 2.16 (1.88, 2.49) | <0.001 | 3.74 (3.17) |
| P for trend | |  |  | <0.001 |  | <0.001 |  | <0.001 |  | <0.001 |  |
| Per SD increase | |  | 1.25 (1.22, 1.28) | <0.001 | 1.25 (1.21, 1.28) | <0.001 | 1.21 (1.18, 1.25) | <0.001 | 1.18 (1.15, 1.22) | <0.001 | 1.64 (1.57) |
| **VAI** |  |  |  |  |  |  |  |  |  |  |  |
| Q1 (0.05 to ≤ 1.05) | 328 | 1,299,414 | Reference |  | Reference |  | Reference |  | Reference |  | Reference |
| Q2 (1.05 to ≤ 1.66) | 459 | 1,296,089 | 1.40 (1.22, 1.62) | <0.001 | 1.34 (1.16, 1.54) | <0.001 | 1.30 (1.13, 1.50) | <0.001 | 1.27 (1.10, 1.46) | 0.001 | 1.86 (1.43) |
| Q3 (1.66 to ≤ 2.69) | 644 | 1,291,131 | 1.98 (1.73, 2.26) | <0.001 | 1.83 (1.60, 2.09) | <0.001 | 1.73 (1.51, 1.98) | <0.001 | 1.63 (1.42, 1.86) | <0.001 | 2.64 (2.19) |
| Q4 (2.69 to ≤ 33.94) | 841 | 1,286,773 | 2.59 (2.28, 2.94) | <0.001 | 2.39 (2.11, 2.72) | <0.001 | 2.17 (1.90, 2.47) | <0.001 | 1.95 (1.71, 2.22) | <0.001 |  |
| P for trend | |  |  | <0.001 |  | <0.001 |  | <0.001 |  | <0.001 | 3.31 (2.81) |
| Per SD increase | |  | 1.25 (1.21, 1.28) | <0.001 | 1.24 (1.20, 1.27) | <0.001 | 1.20 (1.17, 1.24) | <0.001 | 1.17 (1.14, 1.21) | <0.001 | 1.62 (1.54) |
| **LAP** |  |  |  |  |  |  |  |  |  |  |  |
| Q1 (-31.39 to ≤ 23.06) | 259 | 1,306,131 | Reference |  | Reference |  | Reference |  | Reference |  | Reference |
| Q2 (23.06 to ≤ 41.88) | 481 | 1,296,463 | 1.87 (1.61, 2.18) | <0.001 | 1.73 (1.48, 2.01) | <0.001 | 1.68 (1.44, 1.96) | <0.001 | 1.60 (1.37, 1.86) | <0.001 | 2.58 (2.08) |
| Q3 (41.88 to ≤ 72.2) | 643 | 1,288,806 | 2.52 (2.18, 2.91) | <0.001 | 2.26 (1.95, 2.62) | <0.001 | 2.13 (1.84, 2.47) | <0.001 | 1.96 (1.69, 2.27) | <0.001 | 3.33 (2.77) |
| Q4 (72.2 to ≤ 762.09) | 889 | 1,282,007 | 3.50 (3.05, 4.02) | <0.001 | 3.18 (2.76, 3.67) | <0.001 | 2.85 (2.47, 3.29) | <0.001 | 2.49 (2.15, 2.87) | <0.001 | 4.42 (3.72) |
| P for trend | |  |  | <0.001 |  | <0.001 |  | <0.001 |  | <0.001 |  |
| Per SD increase | |  | 1.30 (1.26, 1.33) | <0.001 | 1.29 (1.25, 1.33) | <0.001 | 1.25 (1.21, 1.28) | <0.001 | 1.21 (1.17, 1.24) | <0.001 | 1.71 (1.62) |

Abbreviations: HR, hazard ratios; CI, confidence interval; TyG, triglyceride-glucose; AIP, atherogenic index of plasma; TyG-BMI, TyG related to body mass index; TyG-WC, TyG related to waist circumference; TyG-WHtR, TyG related to waist-to-height ratio; CMI, cardiometabolic index; VAI, visceral adiposity index; LAP, lipid accumulation product.

Model 0 was unadjusted.

Model 1 was adjusted for age and sex.

Model 2 was adjusted for age, sex, ethnicity, education, Townsend deprivation index, alcohol consumption status, smoking status, and physical activity.

Model 3 was adjusted for age, sex, ethnicity, education, Townsend deprivation index, alcohol consumption status, smoking status, physical activity, body mass index (only for TyG index and AIP index), diabetes, cholelithiasis, and lipid-lowering drug.

^a^ P for trend was estimated using the median value of each category.

# Table S5. Intraclass correlation coefficient between two repeated measurements of triglyceride-related indices.

| **Index** | **ICC (95% CI)** |
| --- | --- |
| TyG | 0.63 (0.62, 0.64) |
| TyG-BMI | 0.88 (0.88, 0.89) |
| TyG-WC | 0.83 (0.83, 0.84) |
| TyG-WHtR | 0.81 (0.80, 0.81) |
| AIP | 0.74 (0.73, 0.74) |
| CMI | 0.69 (0.68, 0.70) |
| VAI | 0.65 (0.64, 0.66) |
| LAP | 0.68 (0.67, 0.69) |

Abbreviations: TyG, triglyceride-glucose; TyG-BMI, TyG related to body mass index; TyG-WC, TyG related to waist circumference; TyG-WHtR, TyG related to waist-to-height ratio; AIP, atherogenic index of plasma; CMI, cardiometabolic index; VAI, visceral adiposity index; LAP, lipid accumulation product.

# Table S6. Sensitivity analysis shows the robust association between the triglyceride-related indices with the risk of acute pancreatitis (excluding participants diagnosed with acute pancreatitis within the first two years after baseline).

|  | **Events (n)** | **Person-years** |  | **Model 3** | |
| --- | --- | --- | --- | --- | --- |
|  |  |  |  | **HR (95% CI)** | **P value** |
| **TyG** |  |  |  |  |  |
| Q1 | 297 | 1,305,571 |  | Reference | Reference |
| Q2 | 447 | 1,294,558 |  | 1.22 (1.05, 1.41) | 0.009 |
| Q3 | 581 | 1,289,548 |  | 1.37 (1.19, 1.59) | <0.001 |
| Q4 | 704 | 1,281,736 |  | 1.47 (1.27, 1.70) | <0.001 |
| P for trend | |  |  |  | <0.001 |
| Per SD increase | |  |  | 1.14 (1.08, 1.19) | <0.001 |
| **AIP** |  |  |  |  |  |
| Q1 | 298 | 1,301,123 |  | Reference | Reference |
| Q2 | 427 | 1,295,412 |  | 1.16 (1.00, 1.35) | 0.047 |
| Q3 | 584 | 1,289,600 |  | 1.37 (1.18, 1.58) | <0.001 |
| Q4 | 720 | 1,285,279 |  | 1.52 (1.31, 1.76) | <0.001 |
| P for trend | |  |  |  | <0.001 |
| Per SD increase | |  |  | 1.19 (1.13, 1.25) | <0.001 |
| **TyG-BMI** |  |  |  |  |  |
| Q1 | 263 | 1,303,182 |  | Reference | Reference |
| Q2 | 390 | 1,296,797 |  | 1.30 (1.11, 1.52) | 0.001 |
| Q3 | 542 | 1,291,690 |  | 1.67 (1.44, 1.94) | <0.001 |
| Q4 | 834 | 1,279,744 |  | 2.34 (2.03, 2.70) | <0.001 |
| P for trend | |  |  |  | <0.001 |
| Per SD increase | |  |  | 1.33 (1.28, 1.38) | <0.001 |
| **TyG-WC** |  |  |  |  |  |
| Q1 | 236 | 1,310,404 |  | Reference | Reference |
| Q2 | 410 | 1,299,144 |  | 1.57 (1.33, 1.85) | <0.001 |
| Q3 | 560 | 1,290,293 |  | 2.05 (1.75, 2.40) | <0.001 |
| Q4 | 823 | 1,271,572 |  | 2.79 (2.38, 3.27) | <0.001 |
| P for trend | |  |  |  | <0.001 |
| Per SD increase | |  |  | 1.41 (1.34, 1.48) | <0.001 |
| **TyG-WHtR** |  |  |  |  |  |
| Q1 | 217 | 1,309,249 |  | Reference | Reference |
| Q2 | 421 | 1,299,075 |  | 1.69 (1.43, 2.00) | <0.001 |
| Q3 | 530 | 1,290,296 |  | 1.96 (1.66, 2.31) | <0.001 |
| Q4 | 861 | 1,272,792 |  | 2.84 (2.42, 3.32) | <0.001 |
| P for trend | |  |  |  | <0.001 |
| Per SD increase | |  |  | 1.39 (1.33, 1.45) | <0.001 |
| **CMI** |  |  |  |  |  |
| Q1 | 262 | 1,303,562 |  | Reference | Reference |
| Q2 | 429 | 1,296,025 |  | 1.45 (1.24, 1.69) | <0.001 |
| Q3 | 597 | 1,288,962 |  | 1.85 (1.60, 2.15) | <0.001 |
| Q4 | 741 | 1,282,864 |  | 2.15 (1.85, 2.49) | <0.001 |
| P for trend | |  |  |  | <0.001 |
| Per SD increase | |  |  | 1.18 (1.15, 1.22) | <0.001 |
| **VAI** |  |  |  |  |  |
| Q1 | 295 | 1,299,014 |  | Reference | Reference |
| Q2 | 409 | 1,295,648 |  | 1.26 (1.08, 1.46) | 0.003 |
| Q3 | 583 | 1,290,614 |  | 1.64 (1.43, 1.89) | <0.001 |
| Q4 | 742 | 1,286,137 |  | 1.93 (1.68, 2.22) | <0.001 |
| P for trend | |  |  |  | <0.001 |
| Per SD increase | |  |  | 1.17 (1.14, 1.21) | <0.001 |
| **LAP** |  |  |  |  |  |
| Q1 | 232 | 1,305,760 |  | Reference | Reference |
| Q2 | 430 | 1,296,015 |  | 1.60 (1.36, 1.88) | <0.001 |
| Q3 | 574 | 1,288,264 |  | 1.97 (1.68, 2.30) | <0.001 |
| Q4 | 793 | 1,281,374 |  | 2.51 (2.15, 2.93) | <0.001 |
| P for trend | |  |  |  | <0.001 |
| Per SD increase | |  |  | 1.21 (1.17, 1.25) | <0.001 |

Abbreviations: HR, hazard ratios; CI, confidence interval; TyG, triglyceride-glucose; AIP, atherogenic index of plasma; TyG-BMI, TyG related to body mass index; TyG-WC, TyG related to waist circumference; TyG-WHtR, TyG related to waist-to-height ratio; CMI, cardiometabolic index; VAI, visceral adiposity index; LAP, lipid accumulation product.

Model 3 was adjusted for age, sex, ethnicity, education, Townsend deprivation index, alcohol consumption status, smoking status, physical activity, body mass index (only for TyG index and AIP index), diabetes, cholelithiasis, and using lipid-lowering drug.

# Table S7. Sensitivity analysis shows the robust association between the triglyceride-related indices with the risk of acute pancreatitis (excluding participants with missing covariate data).

|  | **Events (n)** | **Person-years** |  | **Model 3** | |
| --- | --- | --- | --- | --- | --- |
|  |  |  |  | **HR (95% CI)** | **P value** |
| **TyG** |  |  |  |  |  |
| Q1 | 267 | 1,061,489 |  | Reference | Reference |
| Q2 | 385 | 1,032,395 |  | 1.20 (1.03, 1.41) | 0.023 |
| Q3 | 489 | 1,021,028 |  | 1.34 (1.15, 1.56) | <0.001 |
| Q4 | 583 | 1,010,357 |  | 1.41 (1.21, 1.65) | <0.001 |
| P for trend | |  |  |  | <0.001 |
| Per SD increase | |  |  | 1.12 (1.07, 1.18) | <0.001 |
| **AIP** |  |  |  |  |  |
| Q1 | 265 | 1,051,061 |  | Reference | Reference |
| Q2 | 376 | 1,031,235 |  | 1.17 (1.00, 1.38) | 0.048 |
| Q3 | 483 | 1,022,765 |  | 1.30 (1.11, 1.52) | 0.001 |
| Q4 | 600 | 1,020,207 |  | 1.44 (1.23, 1.69) | <0.001 |
| P for trend | |  |  |  | <0.001 |
| Per SD increase | |  |  | 1.17 (1.11, 1.23) | <0.001 |
| **TyG-BMI** |  |  |  |  |  |
| Q1 | 238 | 1,065,597 |  | Reference | Reference |
| Q2 | 359 | 1,049,835 |  | 1.33 (1.13, 1.57) | 0.001 |
| Q3 | 452 | 1,029,299 |  | 1.58 (1.34, 1.85) | <0.001 |
| Q4 | 675 | 980,539 |  | 2.21 (1.89, 2.58) | <0.001 |
| P for trend | |  |  |  | <0.001 |
| Per SD increase | |  |  | 1.30 (1.24, 1.36) | <0.001 |
| **TyG-WC** |  |  |  |  |  |
| Q1 | 203 | 1,057,760 |  | Reference | Reference |
| Q2 | 376 | 1,039,821 |  | 1.68 (1.42, 2.00) | <0.001 |
| Q3 | 458 | 1,027,157 |  | 1.96 (1.64, 2.33) | <0.001 |
| Q4 | 687 | 1,000,531 |  | 2.72 (2.29, 3.24) | <0.001 |
| P for trend | |  |  |  | <0.001 |
| Per SD increase | |  |  | 1.37 (1.30, 1.44) | <0.001 |
| **TyG-WHtR** |  |  |  |  |  |
| Q1 | 191 | 1,076,059 |  | Reference | Reference |
| Q2 | 394 | 1,049,785 |  | 1.83 (1.54, 2.18) | <0.001 |
| Q3 | 447 | 1,026,949 |  | 1.93 (1.62, 2.30) | <0.001 |
| Q4 | 692 | 972,476 |  | 2.75 (2.32, 3.26) | <0.001 |
| P for trend | |  |  |  | <0.001 |
| Per SD increase | |  |  | 1.35 (1.28, 1.42) | <0.001 |
| **CMI** |  |  |  |  |  |
| Q1 | 235 | 1,058,692 |  | Reference | Reference |
| Q2 | 381 | 1,033,776 |  | 1.45 (1.23, 1.71) | <0.001 |
| Q3 | 490 | 1,021,683 |  | 1.71 (1.46, 2.01) | <0.001 |
| Q4 | 618 | 1,011,118 |  | 2.02 (1.72, 2.37) | <0.001 |
| P for trend | |  |  |  | <0.001 |
| Per SD increase | |  |  | 1.17 (1.12, 1.21) | <0.001 |
| **VAI** |  |  |  |  |  |
| Q1 | 257 | 1,060,494 |  | Reference | Reference |
| Q2 | 375 | 1,037,201 |  | 1.35 (1.15, 1.58) | <0.001 |
| Q3 | 490 | 1,023,558 |  | 1.62 (1.39, 1.88) | <0.001 |
| Q4 | 602 | 1,004,017 |  | 1.86 (1.60, 2.16) | <0.001 |
| P for trend | |  |  |  | <0.001 |
| Per SD increase | |  |  | 1.16 (1.12, 1.20) | <0.001 |
| **LAP** |  |  |  |  |  |
| Q1 | 214 | 1,065,212 |  | Reference | Reference |
| Q2 | 379 | 1,037,136 |  | 1.56 (1.32, 1.85) | <0.001 |
| Q3 | 488 | 1,019,443 |  | 1.86 (1.57, 2.19) | <0.001 |
| Q4 | 643 | 1,003,478 |  | 2.27 (1.93, 2.68) | <0.001 |
| P for trend | |  |  |  | <0.001 |
| Per SD increase | |  |  | 1.18 (1.14, 1.23) | <0.001 |

Abbreviations: HR, hazard ratios; CI, confidence interval; TyG, triglyceride-glucose; AIP, atherogenic index of plasma; TyG-BMI, TyG related to body mass index; TyG-WC, TyG related to waist circumference; TyG-WHtR, TyG related to waist-to-height ratio; CMI, cardiometabolic index; VAI, visceral adiposity index; LAP, lipid accumulation product.

Model 3 was adjusted for age, sex, ethnicity, education, Townsend deprivation index, alcohol consumption status, smoking status, physical activity, body mass index (only for TyG index and AIP index), diabetes, cholelithiasis, and using lipid-lowering drug.

# Table S8. Sensitivity analysis shows the robust association between the triglyceride-related indices with the risk of acute pancreatitis (further adjusted for c-reactive protein).

|  | **Further adjusted for C-reactive protein** | |
| --- | --- | --- |
|  | **HR (95% CI)** | **P value** |
| **TyG** |  |  |
| Q1 | Reference | Reference |
| Q2 | 1.21 (1.05, 1.39) | 0.009 |
| Q3 | 1.29 (1.12, 1.48) | <0.001 |
| Q4 | 1.37 (1.18, 1.58) | <0.001 |
| P for trend |  | <0.001 |
| Per SD increase | 1.10 (1.05, 1.15) | <0.001 |
| **AIP** |  |  |
| Q1 | Reference | Reference |
| Q2 | 1.09 (0.94, 1.27) | 0.256 |
| Q3 | 1.22 (1.04, 1.43) | 0.015 |
| Q4 | 1.32 (1.10, 1.57) | 0.002 |
| P for trend |  | <0.001 |
| Per SD increase | 1.15 (1.08, 1.22) | <0.001 |
| **TyG-BMI** |  |  |
| Q1 | Reference | Reference |
| Q2 | 1.25 (1.07, 1.45) | 0.004 |
| Q3 | 1.52 (1.31, 1.76) | <0.001 |
| Q4 | 2.03 (1.75, 2.35) | <0.001 |
| P for trend |  | <0.001 |
| Per SD increase | 1.27 (1.22, 1.32) | <0.001 |
| **TyG-WC** |  |  |
| Q1 | Reference | Reference |
| Q2 | 1.54 (1.32, 1.80) | <0.001 |
| Q3 | 1.85 (1.58, 2.17) | <0.001 |
| Q4 | 2.48 (2.11, 2.92) | <0.001 |
| P for trend |  | <0.001 |
| Per SD increase | 1.34 (1.28, 1.41) | <0.001 |
| **TyG-WHtR** |  |  |
| Q1 | Reference | Reference |
| Q2 | 1.66 (1.41, 1.94) | <0.001 |
| Q3 | 1.81 (1.54, 2.13) | <0.001 |
| Q4 | 2.53 (2.16, 2.97) | <0.001 |
| P for trend |  | <0.001 |
| Per SD increase | 1.32 (1.26, 1.39) | <0.001 |
| **CMI** |  |  |
| Q1 | Reference | Reference |
| Q2 | 1.34 (1.15, 1.57) | <0.001 |
| Q3 | 1.63 (1.39, 1.92) | <0.001 |
| Q4 | 1.85 (1.54, 2.22) | <0.001 |
| P for trend |  | <0.001 |
| Per SD increase | 1.12 (1.08, 1.16) | <0.001 |
| **VAI** |  |  |
| Q1 | Reference | Reference |
| Q2 | 1.17 (1.01, 1.36) | 0.037 |
| Q3 | 1.44 (1.23, 1.68) | <0.001 |
| Q4 | 1.64 (1.38, 1.94) | <0.001 |
| P for trend |  | <0.001 |
| Per SD increase | 1.10 (1.06, 1.14) | <0.001 |
| **LAP** |  |  |
| Q1 | Reference | Reference |
| Q2 | 1.51 (1.30, 1.77) | <0.001 |
| Q3 | 1.78 (1.53, 2.08) | <0.001 |
| Q4 | 2.17 (1.85, 2.54) | <0.001 |
| P for trend |  | <0.001 |
| Per SD increase | 1.15 (1.11, 1.20) | <0.001 |

Abbreviations: HR, hazard ratios; CI, confidence interval; TyG, triglyceride-glucose; AIP, atherogenic index of plasma; TyG-BMI, TyG related to body mass index; TyG-WC, TyG related to waist circumference; TyG-WHtR, TyG related to waist-to-height ratio; CMI, cardiometabolic index; VAI, visceral adiposity index; LAP, lipid accumulation product.

Further adjusted for C-reactive protein based on Model 3.

# Supplementary Figures

# Figure S1. Correlation matrix for triglyceride-related indices, using Pearson’s correlation.

Abbreviations: TyG, triglyceride-glucose; AIP, atherogenic index of plasma; TyG-BMI, TyG related to body mass index; TyG-WC, TyG related to waist circumference; TyG-WHtR, TyG related to waist-to-height ratio; CMI, cardiometabolic index; VAI, visceral adiposity index; LAP, lipid accumulation product.

# Figure S2. Subgroup analysis of TyG index and acute pancreatitis.

# Figure S3. Subgroup analysis of AIP index and acute pancreatitis.

# Figure S4. Subgroup analysis of TyG-BMI index and acute pancreatitis.

# Figure S5. Subgroup analysis of TyG-WC index and acute pancreatitis.

# Figure S6. Subgroup analysis of TyG-WHtR index and acute pancreatitis.

# Figure S7. Subgroup analysis of CMI index and acute pancreatitis.

# Figure S8. Subgroup analysis of VAI index and acute pancreatitis.

# Figure S9. Subgroup analysis of LAP index and acute pancreatitis.

**References**

(1)	Jordan H, Roderick P, Martin D The Index of Multiple Deprivation 2000 and accessibility effects on health. Journal of epidemiology and community health 2004; 58(3): 250-257.

(2)	Simental-Mendía LE, Rodríguez-Morán M, Guerrero-Romero F The product of fasting glucose and triglycerides as surrogate for identifying insulin resistance in apparently healthy subjects. Metabolic syndrome and related disorders 2008; 6(4): 299-304.

(3)	Er LK, Wu S, Chou HH, et al. Triglyceride Glucose-Body Mass Index Is a Simple and Clinically Useful Surrogate Marker for Insulin Resistance in Nondiabetic Individuals. PloS one 2016; 11(3): e0149731.

(4)	Lim J, Kim J, Koo SH, et al. Comparison of triglyceride glucose index, and related parameters to predict insulin resistance in Korean adults: An analysis of the 2007-2010 Korean National Health and Nutrition Examination Survey. PloS one 2019; 14(3): e0212963.

(5)	Onat A, Can G, Kaya H, et al. "Atherogenic index of plasma" (log10 triglyceride/high-density lipoprotein-cholesterol) predicts high blood pressure, diabetes, and vascular events. Journal of clinical lipidology 2010; 4(2): 89-98.

(6)	Wakabayashi I, Daimon T The "cardiometabolic index" as a new marker determined by adiposity and blood lipids for discrimination of diabetes mellitus. Clinica chimica acta; international journal of clinical chemistry 2015; 438: 274-278.

(7)	Amato MC, Giordano C, Galia M, et al. Visceral Adiposity Index: a reliable indicator of visceral fat function associated with cardiometabolic risk. Diabetes care 2010; 33(4): 920-922.

(8)	Taverna MJ, Martínez-Larrad MT, Frechtel GD, et al. Lipid accumulation product: a powerful marker of metabolic syndrome in healthy population. European journal of endocrinology 2011; 164(4): 559-567.
